# Mammalian Kinesin-3 Motors Are Dimeric In Vivo and Move by Processive Motility upon Release of Autoinhibition

**DOI:** 10.1371/journal.pbio.1000072

**Published:** 2009-03-31

**Authors:** Jennetta W Hammond, Dawen Cai, T. Lynne Blasius, Zhe Li, Yuyang Jiang, Gloria T Jih, Edgar Meyhofer, Kristen J Verhey

**Affiliations:** 1 Cell and Developmental Biology, University of Michigan, Ann Arbor, Michigan, United States of America; 2 Biophysics Research Division, University of Michigan, Ann Arbor, Michigan, United States of America; 3 Mechanical Engineering, University of Michigan, Ann Arbor, Michigan, United States of America; Adolf-Butenandt-Institut Zellbiologie, Germany

## Abstract

Kinesin-3 motors drive the transport of synaptic vesicles and other membrane-bound organelles in neuronal cells. In the absence of cargo, kinesin motors are kept inactive to prevent motility and ATP hydrolysis. Current models state that the Kinesin-3 motor KIF1A is monomeric in the inactive state and that activation results from concentration-driven dimerization on the cargo membrane. To test this model, we have examined the activity and dimerization state of KIF1A. Unexpectedly, we found that both native and expressed proteins are dimeric in the inactive state. Thus, KIF1A motors are not activated by cargo-induced dimerization. Rather, we show that KIF1A motors are autoinhibited by two distinct inhibitory mechanisms, suggesting a simple model for activation of dimeric KIF1A motors by cargo binding. Successive truncations result in monomeric and dimeric motors that can undergo one-dimensional diffusion along the microtubule lattice. However, only dimeric motors undergo ATP-dependent processive motility. Thus, KIF1A may be uniquely suited to use both diffuse and processive motility to drive long-distance transport in neuronal cells.

## Author Summary

Molecular motors transport a wide variety of cellular cargoes that are important for diverse cellular phenomena such as mitosis, polarity, motility, and secretion. Motor activity must be tightly regulated to ensure that ATP hydrolysis and processive motility occur only upon coupling to the correct cargo. In neuronal cells, Kinesin-3 motors drive the transport of presynaptic vesicles and other membrane-bound organelles along microtubule tracks. Yet the mechanisms of Kinesin-3 motor activation and motility remain controversial. In this study, we examine the regulation and motile properties of the Kinesin-3 motor KIF1A. We show that in the absence of cargo, KIF1A motors exist in a dimeric inactive state that is maintained by two distinct autoinhibitory mechanisms. This suggests a simple model for activation of dimeric motors upon cargo binding. We also show that dimeric motors can undergo two mechanisms of motility along microtubule tracks: one-dimensional diffusion and ATP-driven processive motility. This unique property may facilitate the ability of KIF1A to drive long-distance vesicular transport in neuronal cells.

## Abstract 3

This study defines the mechanisms of activation and motility of KIF1A motors, which drive the transport of presynaptic vesicles in neuronal cells, and suggests a simple model whereby cargo binding activates dimeric, autoinhibited motors.

## Introduction

Kinesin motors drive the long-distance transport of membrane-bound cargoes along microtubules. Long-distance transport is particularly important in neuronal cells whose length and polarity require robust sorting and transport of cargoes to pre- and postsynaptic destinations. Transport of synaptic vesicle precursors to axon terminals is driven by members of the Kinesin-3 family, the mammalian KIF1A, and Caenorhabditis elegans Unc104 motors [1]. Loss of KIF1A or Unc104 function results in decreased synaptic vesicles in axonal growth cones, and early death [1]. Thus, understanding how kinesin motors are regulated to enable transport of the correct cargo to the proper cellular destination at the relevant time is an important biological problem.

In the absence of cargo, kinesin motors are kept inactive to prevent futile ATP (adenosine triphosphate) hydrolysis and motility. Two models have been proposed for how activity is suppressed in the absence of cargo. The first model posits that dimeric motors are regulated by an autoinhibitory mechanism. Autoinhibition typically involves a folded state that enables the motor's own tail domain to interact with and inhibit its motor domain. This model is based on a large body of work on the Kinesin-1 motor (formerly conventional kinesin or KIF5) [2–5]. In recent years, this model has received increasing experimental support from studies on kinesin motors involved in diverse functions such as epithelial polarity, intraflagellar transport, and mitosis [6–8]. Interestingly, autoinhibition may be a general model for motor regulation, as two well-studied members of the myosin family, nonmuscle myosin II and myosin V, exist in a folded inactive state [9–11]. Autoinhibition enables precise spatial and temporal regulation of motors and may be relieved by cargo binding [6,12], phosphorylation [8], or other mechanisms.

The second model states that motor activity is regulated by transition from a monomeric to dimeric state. Evidence for this model comes from studies on KIF1A/Unc104 motors where the full-length motors exist in a monomeric, inactive state [13–15]. Unc104 activity can be increased by forced dimerization or by an increase in the local concentration of the motor on liposomes [16–18]. Thus, cargo-induced dimerization would enable KIF1A/Unc104 motors to coordinate their two motor domains and step processively in a “hand-over-hand” fashion [19,20]. The cargo-induced dimerization model has gained support from recent studies on the myosin family member myosin VI [21–24]. In this case, the cargo-binding tail domain exists in two mutually exclusive situations: either directly inhibiting the catalytic head in the monomeric state or mediating dimerization on the cargo membrane [25].

The cargo-induced dimerization model for Kinesin-3 motors is primarily based on work with recombinant CeUnc104 proteins. Whether mammalian KIF1A motors are regulated by cargo-induced dimerization has never been tested. In addition, although a region of the KIF1A stalk domain has been shown to inhibit microtubule binding of the motor domain [26], it is unclear how this potential autoinhibitory segment fits in the context of the two models for motor regulation. Furthermore, the sequences that facilitate dimerization remain to be identified.

Here, we test the models for regulation of mammalian KIF1A motors and relate activity to the monomer/dimer state. We show that expressed and endogenous mammalian KIF1A motors exist in a dimeric, inactive state. Thus, cargo-induced dimerization is not a valid model for mammalian KIF1A motors. Rather, we provide support for an autoinhibition mechanism by identifying the sequences and mechanisms required for dimerization and inhibition. Finally, we show that only dimeric motors undergo processive, ATP-driven motility.

## Results

### Expressed KIF1A Motors Are Inactive in Mammalian Cells

**Figure 1:**
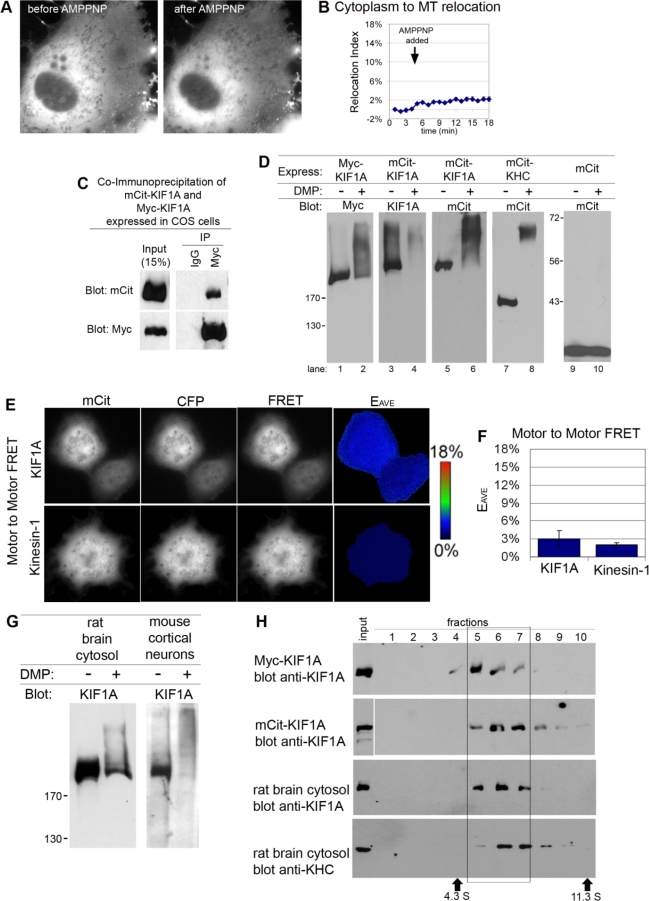
KIF1A Exists as a Dimeric Motor That Is Inactive for Microtubule Binding In Vivo (A and B) Live-cell microtubule binding assay. COS cells expressing mCit-KIF1A were imaged, permeabilized with SLO, and treated with AMPPNP. (A) Still images. (B) Quantification of the percentage of KIF1A-mCit that changed from cytoplasmic to microtubule (MT)-associated localization (Relocation Index) over time. *n* = 16 cells each. (C) Coimmunoprecipitation. Lysates of COS cells coexpressing mCit-KIF1A + Myc-KIF1A were immunoprecipitated (IP) with anti-Myc or control IgG antibodies, separated by SDS-PAGE, and immunoblotted. (D) Crosslinking. Lysates of COS cells expressing Myc-KIF1A, mCit-KIF1A, mCit-KHC, or mCit were untreated or treated with DMP, separated by SDS-PAGE, and immunoblotted. Left, MW standards in kilodaltons. (E and F) Motor-to-motor FRET in live COS cells. (E) Still images of cells coexpressing mCit-KIF1A + mCFP-KIF1A (top panels) or Kinesin-1 (mCit-KHC + mCFP-KHC + HA-KLC; bottom panels). (F) Quantification of *E*_AVE_ (30–40 cells, two to three experiments). (G) Crosslinking. Extracts were treated with or without DMP, separated by SDS-PAGE, and immunoblotted with anti-KIF1A antibodies. Left, MW standards in kilodaltons. (H) Sucrose gradient centrifugation. Fractions were removed from the top (lane 1), run on SDS-PAGE, and immunoblotted. Size standards: catalase (11.3 S) and bovine serum albumin (4.3 S). All data are mean ± SE.

To study the regulation and motile properties of mammalian KIF1A under native conditions, rat KIF1A (Figure S1) was tagged with monomeric citrine (mCit), a variant of yellow fluorescent protein (FP), and expressed in COS cells. This approach has been used successfully to study Kinesin-1 motors and avoids potential problems associated with in vitro purification and/or labeling [2]. In live cells, motor activity can be determined using the nonhydrolyzable ATP analog AMPPNP (5′-adenylyl-beta,gamma-imidodiphosphate) to block the release of active kinesin motors from microtubules [2]. COS cells expressing mCit-KIF1A were transiently permeabilized with the bacterial toxin streptolysin O (SLO). Upon addition of AMPPNP, mCit-KIF1A did not become trapped on microtubules but remained diffuse and cytosolic (Figure 1A and 1B), indicating that KIF1A is not engaged with microtubules when expressed in mammalian cells. Identical results were obtained when the mCit tag was placed at the C-terminus of KIF1A (KIF1A-mCit, Figure S2). The inability to bind microtubules is inherent to KIF1A, as untagged and Myc-tagged versions of KIF1A also did not become trapped on microtubules (Figure S2). In contrast, active versions of Kinesin-1 rapidly became locked on microtubules upon addition of AMPPNP ([2] and Figure S2). We conclude that mammalian KIF1A is inactive in vivo.

### Expressed KIF1A Is a Dimeric Protein

KIF1A could be inactive due to either of the two proposed models for kinesin motor regulation. To distinguish between these possibilities, we first investigated whether expressed KIF1A motors exist as monomers or dimers using coimmunoprecipitation. COS cell lysates coexpressing mCit- and Myc-tagged KIF1A proteins were immunoprecipitated with control antibodies (immunoglobulin G [IgG]) or antibodies to the Myc tag. Coprecipitation of mCit-KIF1A with Myc-KIF1A was observed only with the Myc antibody (Figure 1C), suggesting that KIF1A exists in a dimeric state. As an alternative method, we used chemical crosslinking. Myc- or mCit-tagged versions of KIF1A were expressed in COS cells, and lysates were untreated or treated with dimethylpimilimidate (DMP). In the presence of DMP, both Myc-KIF1A and mCit-KIF1A motors migrated at approximately twice the molecular weight (MW) of the corresponding noncrosslinked proteins (Figure 1D, lanes 1–6). A known dimeric motor, the kinesin heavy chain (KHC) subunit of Kinesin-1, undergoes a similar DMP-induced mobility shift (Figure 1D, lanes 7 and 8). In contrast, mCit showed no shift in mobility upon DMP treatment (Figure 1D, lanes 9 and 10). These results indicate that the expressed KIF1A protein exists in a dimeric state.

We next probed the monomer/dimer state of KIF1A using fluorescence resonance energy transfer (FRET) stoichiometry, a method that calculates an average FRET efficiency (*E*_AVE_) and minimizes effects of variable donor and acceptor FP expression [2,27]. Control experiments show an *E*_AVE_ = 0% for unlinked donor (monomeric cyan FP [mCFP]) and acceptor (mCit) FPs and an *E*_AVE_ = 37% for mCFP and mCit linked by 16 amino acids (unpublished data). For donor and acceptor FPs placed on the N-terminus of KIF1A (“motor-to-motor” FRET), a low but measurable FRET efficiency was obtained (*E*_AVE_ = 3.0 ± 1.2%, *n* = 45 cells, Figure 1E and 1F). This value is not compatible with a monomeric state of the motor. Rather, this value indicates that KIF1A motors exist in a dimeric state in live cells. The motor-to-motor FRET efficiency of KIF1A is comparable to that of the Kinesin-1 holoenzyme (*E*_AVE_ = 2.1 ± 0.4%, *n* = 30 cells, Figure 1E and 1F) whose motor domains are “pushed apart” in the inactive state [2]. By analogy, it is thus possible that the two motor domains of inactive, dimeric KIF1A motors may be pushed apart as part of the regulatory mechanism. FRET stoichiometry was also used to probe the overall conformation of KIF1A. For donor and acceptor FPs localized at the N- and C-termini of a single KIF1A polypeptide (mCit-KIF1A-mCFP), the low but measurable “motor-to-tail” FRET (*E*_AVE_ = 4.8 ± 1.1%, *n* = 39 cells, Figure S3) confirms that KIF1A is not a fully extended molecule. We conclude that KIF1A exists in a compact dimeric state but is not active for microtubule binding.

### Endogenous KIF1A Motors Exist in a Dimeric State

That KIF1A motors expressed under native conditions exist in a dimeric state was surprising as recombinant and endogenous KIF1A/Unc104 motors have been defined as monomeric kinesins based on hydrodynamic analysis. Thus, we tested whether endogenous KIF1A motors also exist in a dimeric state. We first used crosslinking of cytosolic extracts from rat brain and detergent extracts of murine cortical neurons. In the presence of DMP, the endogenous KIF1A proteins showed a reduced mobility (Figure 1G), suggestive of a dimeric state. Crosslinking analysis is dependent on the ability of the antibody to recognize the crosslinked species. Unfortunately, the KIF1A antibody was less able to recognize its antigenic sequence after crosslinking than were antibodies to the epitope tags (Figure 1D). Thus, we sought an alternative method to analyze the monomer/dimer state of endogenous KIF1A motors. Previous reports used hydrodynamic analysis of KIF1A as compared to MW standards [13]. As the shape of kinesin motors may influence their hydrodynamic properties, we sought to compare the sedimentation of endogenous KIF1A motors in sucrose gradients to motors with known oligomeric states. Cytosolic extracts of rat brain or detergent extracts of COS cells expressing dimeric Myc-KIF1A or mCit-KIF1A motors were separated by sucrose gradient sedimentation. The majority of the expressed Myc-KIF1A was found in fractions 5–7 (Figure 1H), whereas the majority of the expressed mCit-KIF1A was found in fractions 6 and 7 (Figure 1H) with the shift likely due to the mCit tag. The endogenous Kinesin-1 protein in rat brain cytosol was also found in fractions 6 and 7 (Figure 1H). Strikingly, the endogenous KIF1A protein in rat brain cytosol was found in fractions 5–7 (Figure 1H), a mobility identical to that of the expressed dimeric Myc-KIF1A and mCit-KIF1A proteins. It is interesting to note that Kinesin-1 (∼360 kDa) and KIF1A motors (∼380–440 kDa) sediment slower than the marker protein catalase (240 kDa) despite their larger size. Thus, sedimentation as compared to MW standards is not a reliable indication of motor mass. Taken together, these results indicate that endogenous KIF1A exists as a dimeric protein.

### Two Autoinhibitory Mechanisms Regulate KIF1A

**Figure 2:**
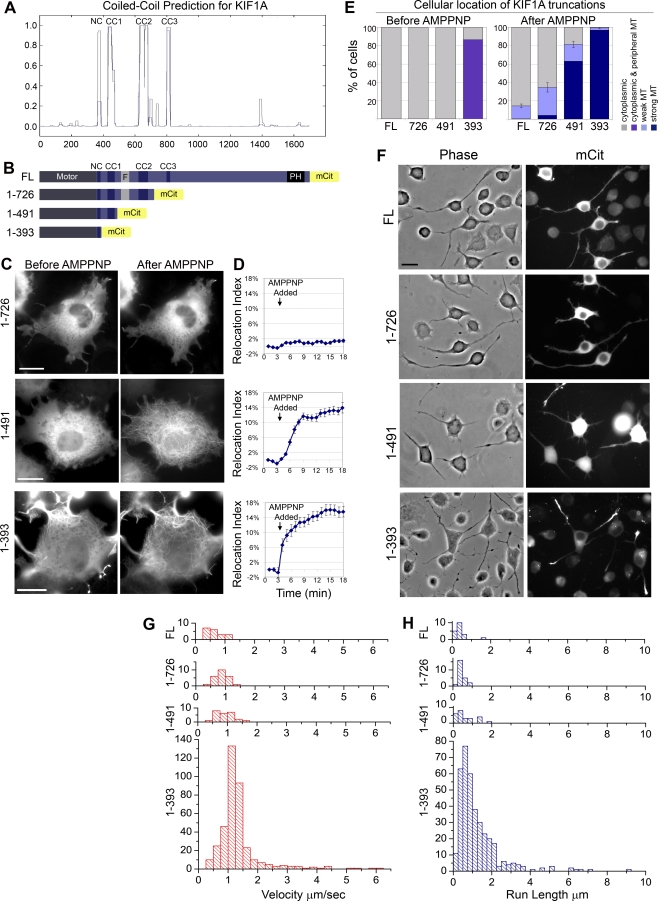
Truncation of the FHA+CC2 Region Relieves Autoinhibition of Microtubule Binding but Not Processive Motility (A) CC prediction for MmKIF1A using windows of 14 (gray line) or 21 (blue line) amino acids (COILS, Lupas Method). (B) Schematic of mCit-tagged FL and truncated KIF1A constructs. (C–E) Live-cell microtubule binding assay. COS cells expressing mCit-tagged KIF1A truncations were imaged during SLO permeabilization and AMPPNP treatment. (C) Still images. Scale bar indicates 20 μm. (D) Quantification of the percentage of KIF1A-mCit that changed from cytoplasmic to microtubule (MT)-associated localization (Relocation Index) over time. (1–726), *n* = 17 cells; (1–491), *n* = 11 cells; (1–393), *n* = 12 cells. (E) Percentage of cells whose KIF1A truncations localize to the indicated cellular locations before and after AMPPNP treatment. (Average = three to five experiments, 50–100 cells each). (F) Processive motility in vivo. Representative images of differentiated CAD cells expressing mCit-tagged constructs. Scale bar indicates 20 μm. (G and H) Processive motility in vitro. Single-molecule motility assays were carried out using COS cell lysates expressing 3xmCit-tagged constructs. Individual spots were tracked, and population velocities (G) and run lengths (H) were plotted as histograms.

That KIF1A exists in a dimeric and inactive state suggests that dimerization is not sufficient for activation. Thus, we tested an autoinhibitory mechanism for KIF1A regulation as related to the domain structure and dimer state of the protein. In KIF1A, a coiled-coil (CC) segment adjacent to the motor domain, referred to as the neck coil (NC), is predicted by the COILS program only if a 14–amino acid window is used for analysis (Figure 2A). The NC is followed by a region of strong coiled-coil prediction (CC1), a forkhead-associated (FHA) domain, and coiled-coil segments CC2 and CC3 (Figure 2A).

Based on this domain structure, we created truncated versions of KIF1A (Figure 2B) by placing a mCit tag after CC2 [KIF1A(1–726)], after CC1 [KIF1A(1–491)], or after the NC [KIF1A(1–393)]. We first tested the ability of these constructs to bind to microtubules. KIF1A(1–726)-mCit remained cytosolic and did not localize to microtubules upon addition of AMPPNP (Figure 2C–2E), indicating that this protein retains the autoinhibited state of the full-length (FL) molecule. In contrast, deletion of the FHA and CC2 segments resulted in a motor, KIF1A(1–491)-mCit, that became trapped in a microtubule-bound state upon addition of AMPPNP (Figure 2C–2E). Thus, the FHA and CC2 domains contribute to autoinhibition by blocking productive interactions with microtubules. This is consistent with previous work on truncated KIF1A/Unc104 motors in vitro [26,28]. Interestingly, deletion of the CC1 domain resulted in a construct, KIF1A(1–393)-mCit, that accumulated on peripheral microtubules at steady state, suggesting that this may be a processive motor. Addition of AMPPNP resulted in an increase in microtubule localization, most notably in the central regions of the cell (Figure 2C). These results suggest that CC1 negatively regulates motility of KIF1A.

To directly analyze the processive motility of FL and truncated KIF1A motors, we used two assays. First, motility in vivo was indirectly assessed by the ability of KIF1A motors to accumulate at the tips of neurites in neuron-like CAD cells. Second, processive motility in vitro was directly measured using single-molecule motility assays and a total internal reflection fluorescence (TIRF) microscope. For the latter, KIF1A constructs were tagged with three tandem copies of mCit for improved signal and decreased photobleaching and photoblinking [29].

When expressed in differentiated CAD cells, KIF1A-mCit and (1–726)-mCit were diffusely localized in the cell body and did not concentrate at the ends of neuronal processes (Figure 2F). In addition, very few motility events were observed in vitro for KIF1A-3xmCit or (1–726)-3xmCit motors (Figure 2G and 2H, Table 1). These results indicate that truncation of the C-terminal half of KIF1A is not sufficient to relieve autoinhibition of microtubule binding (Figure 2C–2E) or processive motility (Figure 2F–2H). Truncation of the FHA+CC2 region also resulted in little to no processive motility for KIF1A(1–491) in vivo (Figure 2F) or in vitro (Figure 2G and 2H, Table 1). This was surprising as KIF1A(1–491) motors bound to microtubules in vivo (Figure 2C–2E). Thus, the FHA+CC2 region contributes to autoinhibition by blocking microtubule binding, but an additional mechanism(s) controls the ability of the motor to undergo processive motility. This second control mechanism may reside, at least in part, in the CC1 domain as KIF1A(1–393) showed a significantincrease in motility as these motors concentrated at the tips of neurites (Figure 2F) and displayed a large number of processive motility events in vitro (Figure 2G and 2H, Table 1). Analysis of the motile properties of KIF1A(1–393) motors gave an average speed of 1.36 ± 0.04 μm/s and an average run length of 1.24 ± 0.06 μm per event (Table 1), comparable to previous studies [13,14,16]. The few motility events observed for the FL, (1–726), and (1–491) motors occurred with decreased velocity and run lengths as compared to (1–393) and were likely due to a dynamic equilibrium between active and inactive states.

**Table 1:**
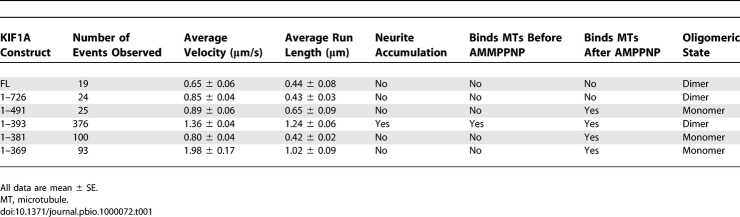
Summary of the Motile Properties and Oligomeric State of FL and Truncated KIF1A Motors

**Figure 3:**
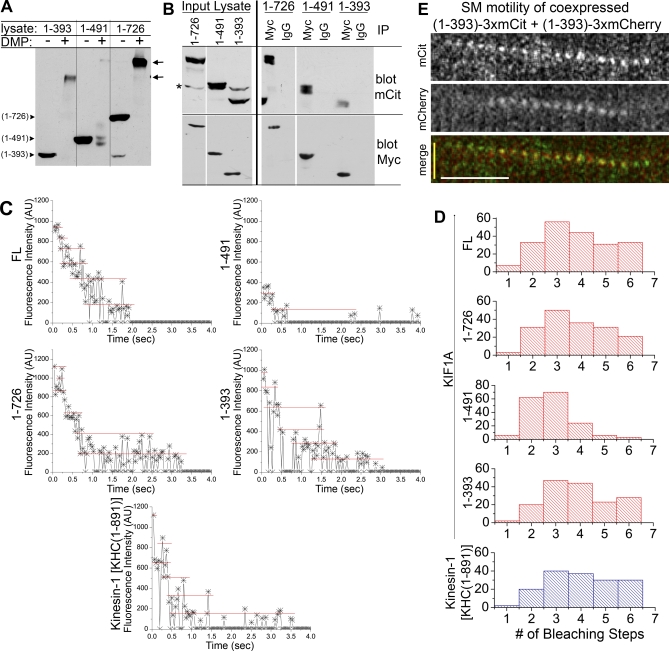
Deletion of CC1 Restores the Dimer State and Processive Motility (A) Crosslinking. Immunoblot of lysates from COS cells expressing the indicated Myc-tagged KIF1A truncations treated with or without DMP. The positions of the truncated proteins in the absence (arrowheads on left) and presence (arrows on right) of DMP are indicated. (B) Coimmunoprecipitation. Lysates of COS cells coexpressing Myc- and mCit-tagged KIF1A constructs of the same length were analyzed directly (input lysate) or after immunoprecipitation (IP) with anti-Myc or control IgG antibodies. Shown are nonadjacent lanes from the same gel. (C and D) Photobleaching analysis. Lysates of COS cells expressing 3xmCit-tagged KIF1A or Kinesin-1 constructs were imaged by TIRF microscopy. (C) Stepwise photobleaching of representative fluorescent spots. AU, arbitrary units. (D) Distribution of the number of photobleaching steps. FL: *n* = 204 traces, average = 3.77 ± 0.10 steps; (1–726): *n* = 173 traces, average = 3.72 ± 0.10 steps; (1–491): *n* = 171 traces, average = 2.83 ± 0.07 steps; (1–393): *n* = 164 traces, average = 3.91 ± 0.10 steps; KHC(1–891): *n* = 159 traces, average = 4.03 ± 0.11 steps. (E) Two-color single-molecule (SM) motility assay. Two-color TIRF imaging of COS cell lysates coexpressing KIF1A(1–393)-3xmCit + KIF1A(1–393)-3xmCherry. Representative time series showing motility of a dual-labeled/dimerized KIF1A(1–393) motor. *y*-axis, distance (yellow bar = 2 μm); *x*-axis, time (white bar = 0.3 s).

Taken together, these results indicate that two regions of KIF1A contribute to autoinhibition. First, the FHA and CC2 domains (amino acids 492–726) prevent microtubule binding (Figure 2), and second, the CC1 domain (amino acids 394–491) inhibits processive motility (Figure 3).

### The CC1 Domain Prevents Processive Motility by Promoting the Monomeric State

To further investigate the relationship between KIF1A domain structure, autoinhibition, and dimerization, we used three assays to test whether truncated motors exist in a dimeric state. We first used chemical crosslinking of KIF1A(1–726)-mCit, KIF1A(1–491)-mCit, and KIF1A(1–393)-mCit motors. In the presence of DMP, most of the (1–726)-mCit and (1–393)-mCit proteins shifted to a higher MW species (Figure 3A), consistent with a dimeric state. In contrast, very little (1–491)-mCit protein displayed a shift in mobility (Figure 3A). Rather, in the presence of DMP, (1–491)-mCit motors showed either the same mobility as the uncrosslinked species or a slightly increased mobility (Figure 3A), perhaps due to an intramolecular crosslink between the CC1 and NC domains [28].

We next used coimmunoprecipitation of Myc- and mCit-tagged truncated KIF1A motors. Coexpression of (1–726)-Myc and (1–726)-mCit resulted in precipitation of (1–726)-mCit by the Myc antibody but not a control antibody (Figure 3B), suggesting that KIF1A(1–726) exists in a dimeric state. Similar results were obtained for the (1–491) and (1–393) truncated motors (Figure 3B). Thus, (1–726) and (1–393) behaved as dimeric proteins by both chemical crosslinking and coimmunoprecipitation, whereas (1–491) showed a more varied behavior.

**Figure 4:**
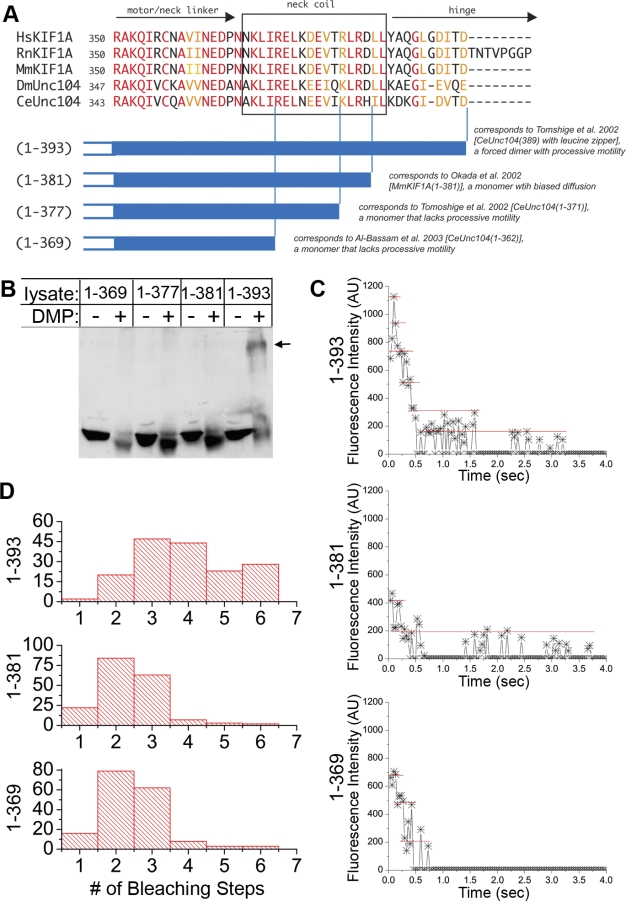
The NC Is Required for Dimerization of KIF1A (A) Sequence comparison (top, ClustalW) of the NC region across species. Red, identical residues; orange, similar residues. Schematic (bottom) of NC constructs used in this study and their relation to KIF1A/Unc104 constructs used in previous studies. (B) Crosslinking. Lysates from COS cells expressing Myc-tagged NC truncations were treated with or without DMP, separated by SDS-PAGE, and immunoblotted with anti-Myc antibodies. Arrow, reduced mobility of KIF1A(1–393) upon crosslinking. (C and D) Photobleaching analysis. Lysates of COS cells expressing 3xmCit-tagged NC constructs were imaged by TIRF microscopy. (C) Stepwise photobleaching of representative spots. (D) Distribution of the number of photobleaching steps. (1–393), *n* = 171 spots, average = 3.91 ± 0.10 steps; (1–381), *n* = 181 spots, average = 2.40 ± 0.07 steps; (1–369), *n* = 171 spots, average = 2.49 ± 0.07 steps.

We then took the advantage of stepwise photobleaching of FPs to test whether truncated versions of KIF1A exist as dimers. This assay has the advantage of analyzing individual motors rather than ensemble averages. Lysates of COS cells expressing 3xmCit-tagged FL or truncated KIF1A motors were analyzed by TIRF microscopy. For each construct, the fluorescence intensity of 160–200 individual fluorescent spots was recorded over time. The number of bleaching steps was determined for each spot (Figure 3C) and then plotted in a histogram to show the population distribution (Figure 3D). In control experiments, the majority of KHC(1–891)-3xmCit motors bleached in four to six steps (Figure 3D and [29]), consistent with the presence of six FPs in the dimeric KHC molecule. A similar distribution of bleaching events was obtained for FL, (1–726), and (1–393) motors (Figure 3D), indicating a dimeric state for these KIF1A constructs. In contrast, the majority of KIF1A(1–491)-3xmCit motors bleached in two to three steps, consistent with a monomeric state (Figure 4D). Taken together, these data suggest that truncated KIF1A constructs that contain only the NC and CC1 domains exist as monomers that are not capable of processive motility.

To directly demonstrate that removal of the CC1 domain restores processive motility as well as the dimeric state, we used two-color TIRF imaging to simultaneously track mCit and mCherry fluorescence in lysates of COS cells coexpressing KIF1A(1–393)-3xmCit and KIF1A(1–393)-3xmCherry. That (1–393) motors move as dimeric molecules is indicated by observations of fluorescent spots labeled with both mCit and mCherry that move together in a linear fashion (representative track, Figure 3E). We also observed mCit and mCherry fluorescent spots with nonoverlapping motility events, as observed when KIF1A(1–393)-3xmCit and KIF1A (1–393)-3xmCherry motors were expressed separately (unpublished data). We believe that these mCit-only and mCherry-only fluorescent spots are also dimeric motors based on their fluorescence intensity. The average maximum mCit fluorescence of mCit-only spots was 513.4 ± 54.0 arbitrary units (*n* = 65 spots). This value is significantly greater than the average maximum mCit fluorescence intensity (356.8 ± 51.4 arbitrary units, *n* = 44 spots) of spots that colabeled and comigrated with mCherry. These results provide the first direct demonstration that KIF1A moves in a directed manner as a dimeric motor.

### Dimerization of KIF1A via the NC Domain

To identify the sequences required for dimerization, we generated KIF1A constructs containing various amounts of the NC region (1–369, 1–377, or 1–381, Figure 4A) or the full NC and several residues of the subsequent hinge region (1–393, Figure 4A). These constructs were designed to directly correspond to previously studied KIF1A motors (Figure 4A). The monomer/dimer state of the NC truncations was tested by crosslinking and photobleaching analysis. In the presence of DMP, the majority of (1–393)-mCit shifted to a higher MW species (Figure 4B). In contrast, (1–381)-mCit, (1–377)-mCit, and (1–369)-mCit showed no mobility change in the presence of DMP (Figure 4B). In photobleaching experiments, a large proportion of KIF1A(1–393)-3xmCit molecules bleached in four to six steps (Figure 4C and 4D), consistent with a dimeric state containing six FPs. However, KIF1A(1–381)-3xmCit and KIF1A(1–369)-3xmCit molecules bleached primarily in two or three steps (Figure 4C and 4D), indicating a monomeric state. These results suggest that the entire NC, as well as residues in the subsequent hinge region, are required for dimerization. This is consistent with a study of synthesized NC peptides where several residues beyond G387 were required to prevent dissociation [30].

### Motile Characteristics of Dimeric KIF1A Motors

**Figure 5:**
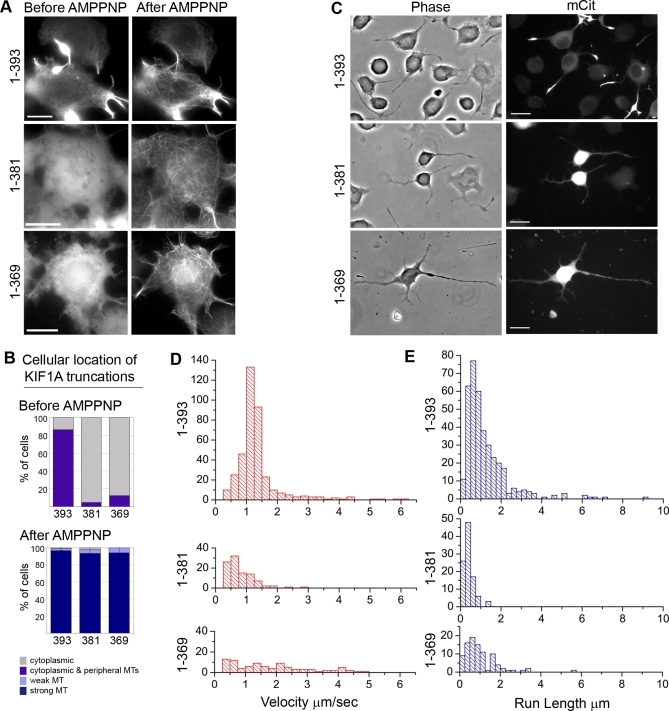
Motor Properties of Monomeric and Dimeric KIF1A Motors (A and B) Live-cell microtubule binding assay. COS cells expressing mCit-tagged NC constructs were imaged during SLO permeabilization and AMPPNP treatment. (A) Representative images before and after AMPPNP treatment. Scale bar indicates 20 μm. (B) Percentage of cells whose NC constructs localize to the indicated cellular locations before and after AMPPNP treatment (averages of two to three experiments, ∼50 cells each). MT, microtubule. (C) In vivo processive motility assays of mCit-tagged NC constructs in differentiated CAD cells. Scale bar indicates 20 μm. (D and E) In vitro single-molecule motility assays of COS cell lysates expressing 3xmCit-tagged NC constructs. (D) Distribution of velocities. (E) Distribution of run lengths.

We next set out to compare the microtubule-based properties of monomeric and dimeric KIF1A motors. We first confirmed that monomeric motors retain the ability to bind to microtubules. Indeed, upon AMPPNP treatment, monomeric (1–381)-mCit and (1–369)-mCit motors became locked on microtubules, similar to dimeric (1–393)-mCit motors (Figure 5A and 5B). We next tested the ability of the NC constructs to undergo processive motility in vivo. The dimeric motor (1–393)-mCit accumulated in neurite tips of differentiated CAD cells, whereas the monomeric motors (1–381)-mCit and (1–369)-mCit remained primarily in the cell bodies (Figure 5C). These results confirm that the full NC is required for dimerization as well as processive motility in mammalian cells.

Finally, we investigated the motile characteristics of (1–393)-3xmCit, (1–381)-3xmCit, and (1–369)-3xmCit motors by in vitro single-molecule motility assays. Motility events that persisted for at least five frames (500 ms) were analyzed to determine velocities and run lengths. As expected, a large number of motility events were observed for dimeric (1–393) motors (Figure 5D and 5E, Table 1). Surprisingly, motility events were also observed for the monomeric (1–381) and (1–369) motors (Figure 5D and 5E, Table 1) even though these motors were not processive in vivo (Figure 5C). However, the motility of the monomeric motors differed from that of the dimeric motor in three ways. First, monomeric motors showed a significant decrease in the number of observed motility events (Table 1). Second, qualitative analysis of the velocity and run-length histograms shows that only dimeric motors gave distributions (Gaussian and single exponential, respectively) characteristic of processive motors (Figure 5D and 5E). Third, dimeric motors underwent significantly longer run lengths (in some cases >6 μm) as dimeric (1–393) motors averaged 1.24 ± 0.06 μm/run, whereas monomeric (1–380) and (1–369) motors averaged only 0.42 ± 0.02 μm/run and 1.02 + 0.09 μm/run, respectively (Figure 5E, Table 1). Thus, monomeric motors are significantly impaired in their motile properties.

### Dimeric but Not Monomeric KIF1A Motors Display ATP-Driven Processive Motility

**Figure 6:**
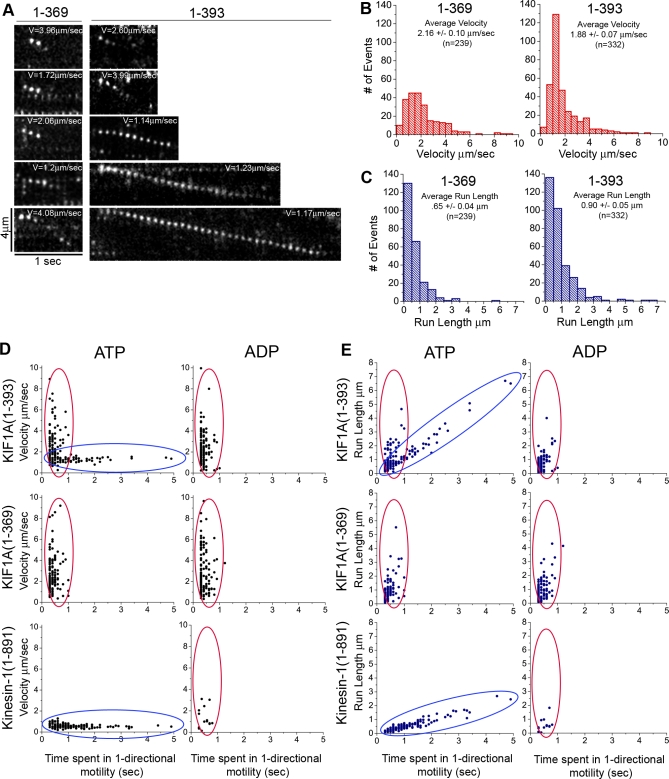
ATP-Dependent Processive Motility Is a Property of Dimeric but Not Monomeric KIF1A Motors In vitro single-molecule motility assays of dimeric (1–393)-3xmCit or monomeric (1–369)-3xmCit KIF1A were carried out. Analysis includes motile events lasting at least 0.3 s. (A) Representative kymographs. (B) Distribution of velocities. (C) Distribution of run lengths. (D and E) Comparison of motile properties of monomeric and dimeric motors in the presence of ATP or ADP. Distribution of velocities (D) or run lengths (E) for KIF1A(1–393), (1–369), or KHC(1–891) in the presence of ATP or ADP as a function of time spent in one-directional motility. Red ovals, 1D diffusion. Blue ovals, processive motility.

We then used two approaches to determine whether the motility of dimeric KIF1A(1–393) motors occurs by one-dimensional (1D) diffusion, as demonstrated for monomeric KIF1A motors, or by ATP-driven processive motility, as demonstrated for other dimeric kinesin motors [19,20]. Single-molecule motility assays were analyzed to include fluorescent spots visible and motile for at least three frames (300 ms) to ensure inclusion of diffusive events (representative times series, Figure 6A). This resulted in an expected decrease in the average run lengths of both dimeric (1–393)-3xmCit and monomeric (1–369)-3xmCit motors (0.90 ± 0.05 μm/run and 0.65 ± 0.04 μm/run, respectively, Figure 6C) as well as increases in the average velocities (1.88 ± 0.07 μm/s and 2.16 ± 0.10 μm/s, respectively, Figure 6B).

Our first approach was to compare the biophysical properties of a large number of motility events by plotting the data as a comparison of the velocities or run lengths against the duration of each motility event (time spent in one-directional motion). By this analysis, drastic differences between monomeric and dimeric mechanisms of motility became apparent. The motility events of dimeric (1–393)-3xmCit motors could be segregated into two classes: first, motile events lasting for short periods of time (<1 s) at a wide variety of speeds and distances (Figure 6D and 6E, top left panels, red circles) and second, motile events lasting for longer time periods (>1 s) at constant speeds of ∼1.2 μm/s (Figure 6D, top left panel, blue circle) and with run lengths directly dependent on the amount of time spent in motion (Figure 6E, top left panel, blue circle). We hypothesized that the first class of motility (Figure 6D and 6E, red circles) is 1D diffusion, whereas the second class of motility (Figure 6D and 6E, blue circles) is processive motility. Indeed, monomeric KIF1A(1–369)-3xmCit motors only moved for short periods of time (<1 s) at a wide variety of speeds and distances (Figures 6D and 6E, middle left panels, red circles), indicative of 1D diffusion. In contrast, the processive motility of dimeric KHC(1–891)-3xmCit motors was evident as these motors spent longer periods of time in motion (>1 s) at a constant speed (Figure 6D, bottom left panel, blue circle) and for distances directly dependent on the time spent in motion (Figure 6E, bottom left panel, blue circle). Thus, dimeric KIF1A(1–393) motors display motility properties of both 1D diffusion and processive motility.

Our second approach was to distinguish these motility classes by their ATP dependence. Diffusion of monomeric KIF1A motors occurs in the weakly bound or ADP (adenosine diphosphate) state [31], whereas processive hand-over-hand stepping of kinesin motors requires the energy of ATP hydrolysis. When single-molecule motility assays of monomeric (1–369)-3xmCit motors were carried out in the presence of ADP, no change in motility was observed. Monomeric motors continued to move only for short periods of time (<1 s) at various speeds and run lengths (Figure 6D and 6E, middle right panels, red circles), confirming that monomeric KIF1A(1–369)-3xmCit motors moved by 1D diffusion. In contrast, the processive motility of dimeric KHC(1–891)-3xmCit motors was abolished in the presence of ADP (Figures 6D and 6E, bottom right panels). The motile properties of dimeric (1–393)-3xmCit motors were also highly dependent on nucleotide. Dimeric (1–393)-3xmCit motors continued to display short motility events (<1 s) of various velocities and run lengths (Figure 6D and 6E, top right panels, red circles) in the presence of ADP, similar to the monomeric motors. However, dimeric motors were unable to undergo processive motility in the presence of ADP (Figure 6D and 6E, top right panels). This analysis demonstrates that for motility events that last only short time periods, it is not possible to distinguish 1D diffusion from processive, hand-over-hand motor stepping (overlap of red and blue circles). However, for events that last longer than 0.5–1 s, comparisons of velocity and run length to time spent in directional motility enable the separation of motility mechanisms. We conclude that KIF1A motors exist as dimeric molecules that can move by 1D diffusion but show processive motility only in the presence of ATP.

## Discussion

The motile properties of kinesin motors must be tightly coupled to the binding and transport of cargo. Two models have been proposed to account for the inactive state of kinesin motors in the absence of cargo. We show that the current model for Kinesin-3 motors, that inactive monomeric motors are activated by cargo-induced dimerization, is not valid for KIF1A as expressed and endogenous motors exist in a dimeric and inactive state. We show instead that regulation is due to autoinhibition by nonmotor regions. Thus, autoinhibition of dimeric motors appears to be a general mechanism for regulating molecular motors.

### Dimerization of KIF1A

We provide four lines of experimental evidence (chemical crosslinking, coimmunoprecipitation, FRET, and photobleaching) to demonstrate that FL KIF1A expressed in mammalian cells exists as a dimeric protein. A concentration-dependent push to the dimeric state seems unlikely since crosslinking, coimmunoprecipitation, and photobleaching assays are carried out under dilute conditions. We also show that endogenous KIF1A behaves as a dimeric motor by crosslinking and sucrose gradient sedimentation. The sedimentation behavior of endogenous KIF1A was previously interpreted, based on comparisons to size standards, as indicating a protein of approximately 200 kDa and thus a monomeric molecule [13]. However, we show that the sedimentation behavior of endogenous KIF1A is consistent with a dimeric state when compared to Kinesin-1 and KIF1A motors whose oligomeric state has been confirmed in alternative assays.

The sedimentation behavior of KIF1A is not surprising considering the behavior of other kinesin motors in these assays. Values of 6.7 S have been reported for Kinesin-1 (∼340 kDa) [32], 8.6–9.8 S for the heterotrimeric Kinesin-2 motor KIF3A/KIF3B/KAP (∼300 kDa) [33,34], and 6.8–7.9 S for the homodimeric Kinesin-2 motor CeOSM-3 (∼160 kDa) [7,34]. Thus, the oligomeric state of kinesin motors cannot be conclusively determined in sucrose gradients, and perhaps not in other biochemical analyses, by using MW standards and without considering other variables such as protein conformation. In conclusion, our data provide strong support for the idea [6,35] that, like Kinesin-1 and Kinesin-2 motors, Kinesin-3 family members are dimeric motors.

Our results further show that the NC plays an important role in mediating dimerization. This is consistent with studies showing coil formation by peptides corresponding to the predicted NC regions of MmKIF1A and CeUnc104 [15,30]. Importantly, we show that several residues in the hinge segment C-terminal to the NC are required for dimerization and processive motility, providing an explanation for why previous constructs that lack a full NC resulted in monomeric motors [16,28,31,36,37]. A role for the hinge region in dimerization and/or processive motility has been noted for other kinesins, most notably fungal Kinesin-1 [38].

### Autoinhibition of KIF1A

Our results demonstrate that KIF1A motors can exist in a dimeric but autoinhibited state. These results do not support a model for cargo-mediated dimerization of Kinesin-3 motors but rather suggest a simple model in which cargo binding relieves autoinhibition of dimeric motors. Using truncation mutants, we identified two regions that contribute to different mechanisms of autoinhibition. First, the FHA and CC2 domains inhibit the interaction of KIF1A with microtubules as KIF1A(1–491) motors can bind to microtubules but cannot undergo processive motility, consistent with previous work [39]. Second, the CC1 domain regulates processive motility as KIF1A(1–393) motors can both bind to microtubules and undergo processive motility. We show that CC1 blocks processive motility by interference with the formation of dimeric motors, perhaps due to an intramolecular interaction with the NC as seen in truncated CeUnc104 motors [28]. This interaction may contribute to the altered mobility of (1–491) motors upon crosslinking. Further studies are required to test whether CC1-mediated inhibition occurs in the FL motor. In this respect, the interaction of CC1 and NC could serve to keep the two motor domains separated and/or uncoordinated, similar to the role of kinesin light chain (KLC) in the autoinhibition of Kinesin-1 [2]. As two separate mechanisms are also involved in the autoinhibition of Kinesin-1 [2], the dual-inhibition mode may be a common way to inhibit the activity of kinesin motors in the absence of cargoes.

Autoinhibition is a common regulatory strategy used in diverse biological systems [40]. In terms of motor regulation, autoinhibition prevents futile ATP hydrolysis and allows rapid and specific control of motor activity both temporally and spatially. Autoinhibition has now been shown to regulate members of the Kinesin-1, Kinesin-2, and Kinesin-3 families (this study and [5–7]). Thus, the evolutionary development of new kinesin families by the attachment of novel cargo-binding domains onto the catalytic kinesin core may have simultaneously ensured that each family adopt a unique mechanism for autoinhibition.

### Mechanisms of KIF1A motility

Our finding that endogenous KIF1A motors exist in a dimeric state indicates that the physiological form that drives processive motion of cargoes in cells is likely to be the dimeric motor. We show that the motility of dimeric KIF1A motors is characterized by smooth, unidirectional movement along microtubules and occurs with an average velocity comparable to vesicles moving in vivo [39,41]. Whether dimeric KIF1A motors utilize a hand-over-hand mechanism like Kinesin-1 is a question for future experiments. That both monomeric and dimeric forms of KIF1A undergo 1D diffusion on microtubules likely indicates a nonspecific binding to the microtubule that enhances processivity.

The ability of dimeric KIF1A motors to undergo both diffusive motion and processive motility is unique among kinesins involved in vesicle transport. Yet, it is interesting that 1D diffusion along microtubules has been reported for all three motor classes. The homotetrameric Kinesin-5 motor Eg5 undergoes ATP-independent 1D diffusion in the absence of cargo and switches to ATP-dependent processive motility in the presence of cargo, resulting in the sliding antiparallel microtubules during formation of the mitotic spindle [42,43]. Similarly, mitotic centromere-associated kinesin (MCAK), a Kinesin-13 motor, uses ATP-independent 1D diffusion to reach microtubule plus ends where ATP-dependent catalytic activity results in depolymerization of microtubules [44]. Surprisingly, Myosin Va can undergo rapid diffusion along the microtubule lattice in vitro [45]. Finally, diffusive motion of the dynactin complex along microtubules may facilitate cytoplasmic dynein processivity [46]. KIF1A may thus be uniquely positioned among vesicular motors to drive long-distance motility using 1D diffusive motion to tether motors to microtubules, as well as ATP hydrolysis for force production.

## Materials and Methods

### Plasmids and antibodies.

FL or truncated rat KIF1A constructs tagged with mCit, 3xmCit, or Myc were generated using convenient restriction sites or PCR and cloned into vectors mCit-N1/C1 (i.e., Clontech's EYFP-N1/C1 vectors), 3xmCit-N1 [29], or pRK5-Myc, respectively. Rat KIF1A(1–393)-mCit was a gift from Gary Banker. All plasmids were verified by DNA sequencing. KHC(1–891)-3xmCit has been described previously [29]. The following antibodies were used: KIF1A (BD Transduction Labs), Myc (Sigma), and GFP (Invitrogen). HA (Covance) and Flag (Sigma) were used as control IgG.

### Cells, transfection, crosslinking, immunoprecipitation, and sucrose density gradient centrifugation.

COS and CAD cells were cultured, transfected, and processed for immunofluorescence or immunoprecipitation as previously described [47]. For crosslinking, cells were lysed in Borate buffer (50 mM NaBorate, 100 mM potassium acetate, 2 mM MgCl_2_, 1 mM EGTA, 1% Triton X-100, and protease inhibitors [pH 8.57]), cleared of insoluble material by centrifugation, then incubated for 30 min with 20 mM DMP (Sigma). An equal volume of 50 mM NH_4_Cl_2_ in PBS was added for 10 min to quench the reaction. Lysates were analyzed by SDS-PAGE and western blot. Sucrose density gradient centrifugation was performed as described [48]. Catalase (11.3 S, 256 kDa; Calbiochem) and bovine serum albumin (4.3 S, 66 kDa; Sigma) were used as standards.

### In vivo microtubule binding and FRET assays.

Microtubule binding and FRET stoichiometry assays in live cells were carried out as described [2]. For microtubule binding in fixed cells, cells were treated with SLO and AMPPNP in the presence of taxol for 10 min and then fixed with 3.7% paraformaldehyde and processed for immunofluorescence. The Relocation Index was calculated on a frame-by-frame basis using ImageJ as described [2].

### In vitro single-molecule motility assays and photobleaching analysis.

Motility and photobleaching assays were performed using a custom objective-type TIRF microscope as described [29]. Briefly, flow chambers were sequentially incubated with Cy5-microtubules, casein, and cell lysates in oxygen-scavenging buffer. Lysates were incubated with ATP or ADP for motility assays or with AMPPNP for photobleaching analysis. The 488-nm line of a tunable, single-mode, fiber-coupled argon ion laser (Melles Griot) was used for excitation. Images were captured every 100 ms. Two-color TIRF was achieved by combining a yellow diode, pumped, solid-state laser (593 nm; CrystaLaser) with the 488-nm laser using a dichroic mirror (Z488RDC). mCit and mCherry fluorescence emissions were first passed though a FF495/605 dual-band dichroic mirror (Semrock) and then projected separately onto each half of the charge-coupled device (CCD) camera by a Dualview beam-splitter (Optic Insights) equipped with a T585LP dichroic beam splitter and ET525/50M and HQ610LP emission filters (Chroma).

All of the fluorescence image analyses were analyzed as described [29] using ImageJ (NIH) and Origin. For single-molecule tracking, measurements for each construct come from at least two independent protein preparations and include motile events lasting at least five frames (500 ms) unless indicated otherwise. All data are presented as mean ± standard error (SE).

Additional methods are available in the supporting information (Text S1).

## Supporting Information

Figure S1 Sequence Comparison of KIF1A Motor Sequences across Species (622 KB TIF)

Figure S2 Full-Length KIF1A Is Inactive for Microtubule Binding Regardless of Epitope Tag (1.52 MB TIF)

Figure S3 Analysis of the Overall Structure of KIF1A by FRET Stoichiometry (27.45 MB TIF)

Text S1 Supplemental Methods (38 KB DOC)

## Abbreviations

1D: one-dimensional
CC: coiled-coil
DMP: dimethylpimilimidate
FHA: forkhead-associated
FL: full-length
FP: fluorescent protein
FRET: fluorescence resonance energy transfer
IgG: immunoglobulin G
KHC: kinesin heavy chain
mCFP: monomeric cyan fluorescent protein
mCit: monomeric citrine
MW: molecular weight
NC: neck coil
TIRF: total internal reflection fluorescence
SLO: streptolysin O
