# Supplementary figures and images for: Mammalian Kinesin-3 Motors Are Dimeric In Vivo and Move by Processive Motility upon Release of Autoinhibition
Source: PLoS Biol. 2009 Mar 31;7(3):e1000072. doi: 10.1371/journal.pbio.1000072 (PMC2661964; doi:10.1371/journal.pbio.1000072)

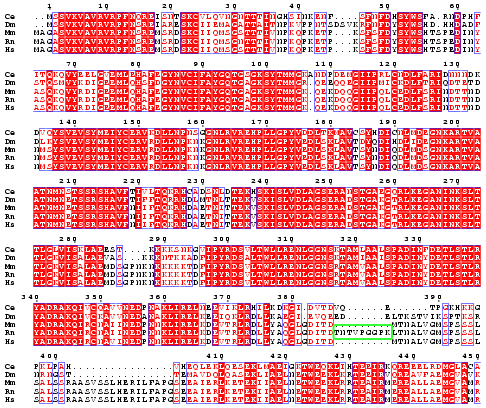

Supplement: Figure S1 — (622 KB TIF) [file pbio.1000072.sg001.tif]

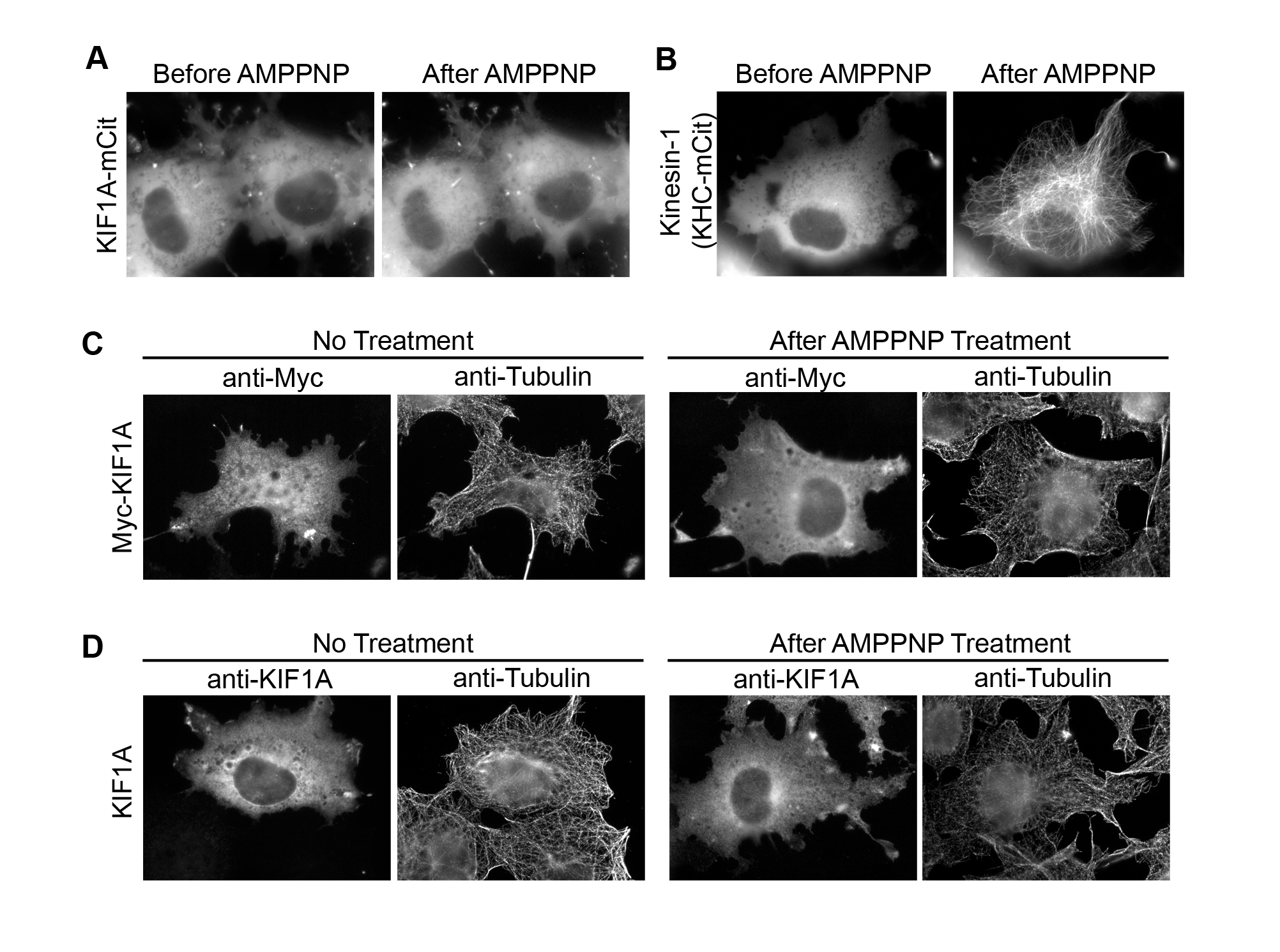

Supplement: Figure S2 — (1.52 MB TIF) [file pbio.1000072.sg002.tif]

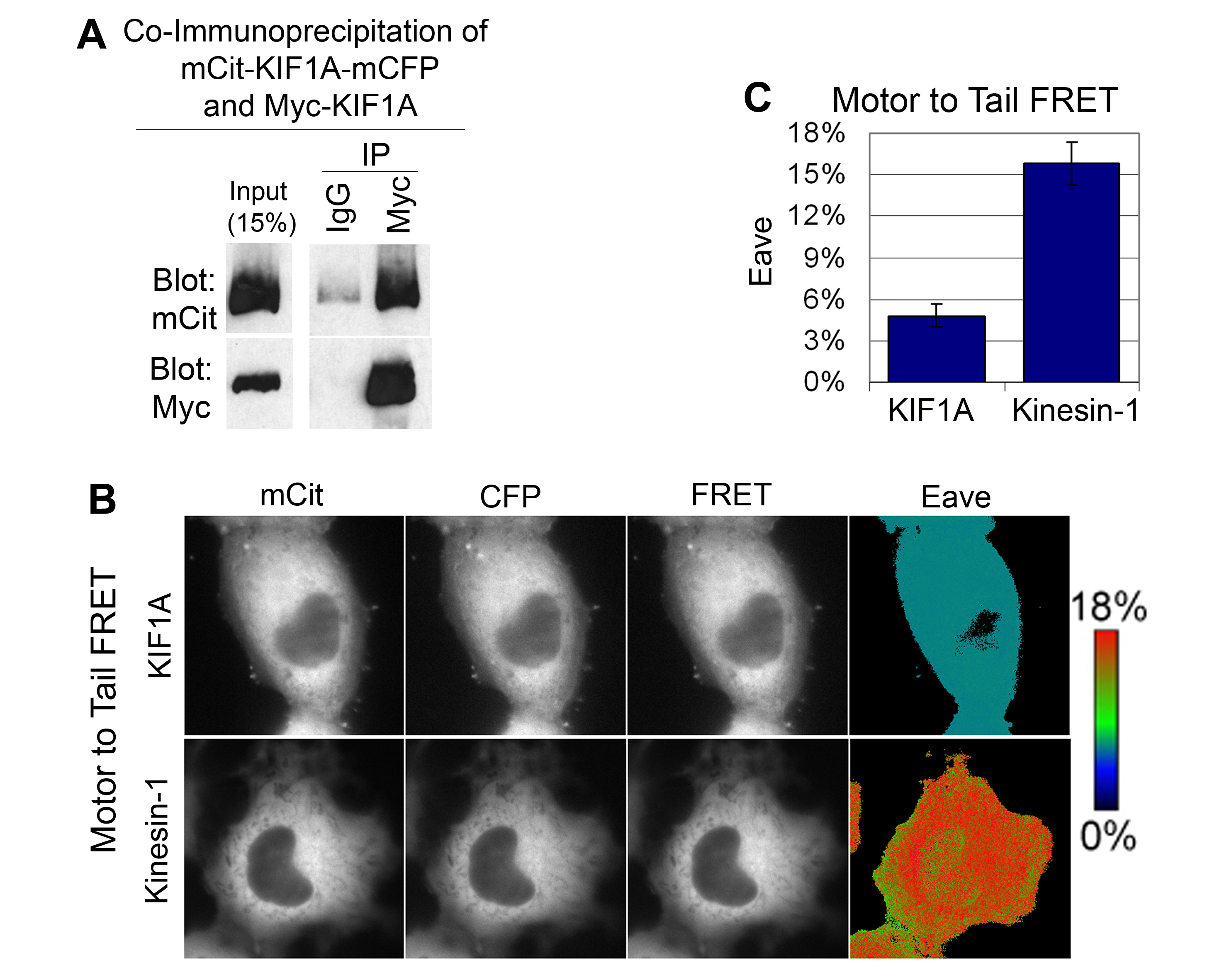

Supplement: Figure S3 — (27.45 MB TIF) [file pbio.1000072.sg003.tif]
