# Supplementary material for: Mammalian Kinesin-3 Motors Are Dimeric In Vivo and Move by Processive Motility upon Release of Autoinhibition
Source: PLoS Biol. 2009 Mar 31;7(3):e1000072. doi: 10.1371/journal.pbio.1000072 (PMC2661964; doi:10.1371/journal.pbio.1000072)
Supplement: Text S1 — (38 KB DOC) [file pbio.1000072.sd001.doc]

**SUPPORTING INFORMATION**

**SUPPLEMENTAL METHODS**

*Immunoprecipitation*. Transfected COS cells were resuspended in lysis buffer (LB; 25 mM Hepes/KOH, 115 mM potassium acetate, 5 mM sodium acetate, 5 mM MgCl_2_, 0.5 mM EGTA, 1% Triton X-100, and protease inhibitors; pH 7.4). Insoluble material was removed by centrifugation at 20,000xg at 4°C for 10 mins and soluble extracts were incubated with the specified antibodies for 2.5-18 hr at 4°C. Protein A agarose beads were added for an additional 30-60 mins at 4°C. Beads were pelleted and washed two times with LB, resuspended in Laemmli sample buffer, and analyzed by SDS-PAGE and western blot.

*Sucrose density gradient centrifugation.* 300 μl 9-17% linear sucrose gradients in LB were prepared at 4°C. 30 μl of COS cell lysate or rat brain cytosol was loaded on the top. Gradients were centrifuged at 38,000 rpm for 4 hrs at 4°C in a Beckman SW 50.1 rotor. Fractions were removed from the top and analyzed by SDS-PAGE and western blot.

*Live cell microtubule binding assay.* COS cells were plated onto glass-bottomed dishes (MatTek) and transfected with plasmids encoding the proteins of interest. 24 hrs later, cells were viewed on a Nikon TE2000 inverted microscope equipped with a Plan-APO 60X/NA 1.4 objective and Photometrics CS ES2 camera. Cells were treated with 0.1 μg/ml Streptolysin O in permeabilization buffer (PB: 25 mM Hepes/KOH, 115 mM potassium acetate, 5 mM sodium acetate, 5 mM MgCl_2_, 0.5 mM EGTA, and 10 mg/ml BSA; pH 7.4) for 1 min. After washing 3 times with PB, cells were incubated with PB containing 2 mM AMPPNP. Cells were monitored every minute for an additional 15 minutes.

*In vitro single molecule motility assays and photobleaching analysis.* Motility and photobleaching assays were performed in flow chambers created from a 25x25 mm, #1.5 coverglass (Corning) and slide (Fisher) assembled with double-sided tape (~30ul chamber volume). Cy5-labeled microtubules in BRB80 buffer (80 mM Pipes/KOH, 1 mM EGTA, 2 mM MgCl_2_; pH 6.8) with 10 μM taxol were flowed into the chamber, followed by addition of 40 μl of 0.1mg/ml casein/BRB80 to coat the chamber surfaces. After 5-10 minutes, chambers were washed twice in P12 buffer (12 mM Pipes/KOH, 1 mM EGTA, 2 mM MgCl_2_, pH 6.8) with 10 µM taxol.

Motor proteins were prepared by lysing transfected COS cells in LB with 1mM ATP. Lysates were cleared by centrifugation at 20,000xg for 10 mins and then flash-frozen by immersion in liquid nitrogen and stored at -80°C. 0.25-1 μl of cell lysate was added to flow-chambers with 50 µl of oxygen scavenger buffer (1 mM DTT, 1 mM MgCl_2_, 2 mM ATP, 10 mM glucose, 0.1 mg/ml glucose oxidase, 0.08 mg/ml catalase, 10 mg/ml BSA, 10 µM taxol in P12). For photobleaching experiments or motility experiments, 2 mM AMPPNP or 2 mM ADP, respectively, were used.

The flow chamber was sealed with wax and assayed for motility or photobleaching using objective-type TIRF microscopy on a custom-modified microscope (Axiovert 135TV; Zeiss) equipped with an 1.45 NA α-plan Fluor objective, 2.5x optovar, 505DCXR dichroic mirror, HQ510LP emission filter, and a back-illuminated EMCCD camera (Cascade 512B; Roper Scientific). The 488-nm line of a tunable, single-mode, fiber-coupled argon ion laser (Melles Griot) was used for excitation. Images were captured every 100 ms for 30-35 sec. The successive positions of individual molecules on a microtubule were linked together into trajectories, or tracked motility events. Only fluorescent spots with a minimum run length of 5 frames (500 ms) were counted in the ensemble analysis except for Figure 6 where a minimum run length of 3 frames (300 ms) was used.

Quantitative analysis to directly compare the motility across KIF1A constructs required the control of two parameters across constructs and experiments. First, the amount of expressed protein was normalized by western blotting with an anti-GFP antibody and second, each 3xmCit-labeled motor was observed for the same total amount of time.

For photobleaching, as each mCit molecule photobleaches, the total fluorescence intensity of the motor construct drops at relatively consistent intervals. Thus, a dimeric 3xmCit-tagged motor could show up to six bleaching steps, whereas, a monomeric 3xmCit-tagged motor could only have up to three steps. Variation in the number of bleaching steps arises from the tendency of FPs to blink, bleach simultaneously, bleach prior to analysis, or fail to mature properly.

**SUPPLEMENTAL FIGURES**

**Figure S1: Sequence comparison of RnKIF1A.** Alignment of the N-terminal regions of rat KIF1A (Rn) with human KIF1A (Hs; NP_004312), Mus musculus KIF1A (Mm; NP_032466), *C elegans* Unc104 (Ce: NP_741019) and *Drosophila* Unc104 (Dm: NP_611155). Note the insertion in RnKIF1A (green box) immediately following the NC. This sequence is identical to that found in a variant of MmKIF1A by Pierce et al. [14].

**Figure S2: Full-length KIF1A is inactive for microtubule binding regardless of epitope tag.**

(A,B) Live cell microtubule binding assay. COS cells expressing (A) KIF1A-mCit or (B) KHC-mCit were imaged before, during and after SLO permeabilization and AMPPNP treatment. Still images are shown for the same cells before and after AMPPNP addition. KIF1A-mCit is diffuse and cytosolic before and after AMPPNP treatment indicating that KIF1A is in an inactive state. In contrast, KHC-mCit becomes trapped on microtubules upon AMPPNP treatment indicating that the KHC subunit is active when expressed in mammalian cells.

(C,D) Live cell microtubule binding assay. To verify that the FP tag did not interfere with the microtubule-binding and/or regulation of KIF1A, we tested the ability of non-tagged and Myc-tagged versions of KIF1A to interact with microtubules. COS cells expressing (C) Myc-KIF1A or (D) untagged KIF1A were permeabilized with SLO and treated with AMPPNP. The cells were then fixed and stained with antibodies to (C) the Myc tag and total tubulin or (D) KIF1A and total tubulin. Neither Myc-KIF1A nor untagged KIF1A localized to microtubules upon addition of AMPPNP. These results indicate that KIF1A is inactive for microtubule binding when expressed in mammalian cells and that the FP tags do not interfere with KIF1A motor function or regulation.

**Figure S3: Analysis of the overall structure of KIF1A by FRET stoichiometry.**

To assess the overall structure of KIF1A molecules expressed in mammalian cells, donor and acceptor FPs were fused to the N- and C-termini of a single KIF1A polypeptide (mCit-KIF1A-mCFP). (A) Dimerization of mCit-KIF1A-mCFP was tested by co-immunoprecipitation. COS cells were co-transfected with plasmids encoding mCit-KIF1A-mCFP and Myc-KIF1A. Cell lysates were then analyzed by western blot either directly (input) or after immunoprecipitation with anti-Myc or control IgG antibodies. The coprecipitation of mCit-KIF1A-mCFP with antibodies to the Myc tag indicates that mCit-KIF1A-mCFP exists in a dimeric state.

(B,C) FRET stoichiometry was then used to test the prediction, based on rotary shadowing of recombinant MmKIF1A [13], that KIF1A in mammalian cells is tightly folded into a compact conformation despite its extended length. (B) “Motor-to-tail” FRET stoichiometry was measured in live COS cells co-expressing mCit-KIF1A-mCFP + Myc-KIF1A (top panels) or Kinesin-1 (mCit-KHC-mCFP + HA-KLC, bottom panels). Still images are shown. (C) A low but detectable motor-to-tail FRET efficiency was obtained for KIF1A (E_AVE_ = 4.8 ± 1.1%, n = 39 cells). This value is lower than the motor-to-tail FRET efficiency of the Kinesin-1 holoenzyme (E_AVE_ = 15.7 ± 1.7%, n = 27 cells) whose motor and tail domains directly interact in the folded inactive state [2-4]. These results indicate that KIF1A expressed under native conditions is not an extended molecule (E_AVE_ ≠ 0). However, a significantly lower motor-to-tail FRET than Kinesin-1 suggests that the KIF1A motor and tail domains do not directly contact each other.
